# Epidemic *Clostridium difficile* Ribotype 027 in Chile

**DOI:** 10.3201/eid1808.120211

**Published:** 2012-08

**Authors:** Cristian Hernández-Rocha, Jonathan Barra-Carrasco, Marjorie Pizarro-Guajardo, Patricio Ibáñez, Susan M. Bueno, Mahfuzur R. Sarker, Ana María Guzman, Manuel Álvarez-Lobos, Daniel Paredes-Sabja

**Affiliations:** Pontificia Universidad Católica de Chile, Santiago, Chile (C. Hernández-Rocha, P. Ibáñez, S.M. Bueno, A.M. Guzman, M. Álvarez-Lobos);; Universidad Andrés Bello, Santiago (J. Barra-Carrasco, M. Pizarro-Guajardo, D. Pareses-Sabja);; and Oregon State University, Corvallis, Oregon, USA (M.R. Sarker, D. Paredes-Sabja)

**Keywords:** *Clostridium difficile*, epidemic strains, severe infection, South America, Chile, bacteria

**To the Editor**: The increased severity of *Clostridium difficile* infection is primarily attributed to the appearance of an epidemic strain characterized as PCR ribotype 027 (*1*). The only report that identified epidemic *C. difficile* ribotype 027 in an American country outside of North America comes from Costa Rica, raising the possibility that strains 027 might also be present in other countries of Latin America (*2*). Several studies between 2001 and 2009 have been conducted in South American countries to detect the incidence of *C. difficile* infection in hospitalized patients, but they did not identify which *C. difficile* strains were causing these infections (*3*).

During an epidemiologic screening of patients with *C. difficile* infection in a university hospital in Chile, we analyzed all stool samples of patients with suspected *C. difficile* infection during a 5-month period (June–November 2011). Two cases of *C. difficile* infection were associated with ribotype 027.

*C. difficile* was isolated from stool samples according to published protocols (*4*). Briefly, stool samples were spread onto taurocholate-cefoxitin-cycloserine fructose (Merck, Rahway, NJ, USA) agar plates and incubated for 96 hours at 37°C in a Bactron III-2 anaerobic workstation (SHEL LAB, Cornelius, OR, USA.). Plates were examined for the characteristic *p*-cresol odor unique to *C. difficile* culture (*5*). The aminopeptidase test (*6*) was also used to differentiate *C. difficile* strains. Suspected colonies were further analyzed by PCR to amplify *tcdA*, and *tcdB* genes (*7*). The presence of binary toxin gene (*cdtB*) and deletion in the negative regulator of the pathogenecity locus, *tcdC*, were determined by using Cepheid GeneXpert PCR (Cepheid, Sunnyvale, CA, USA). We used *C. difficile* ribotype 027 strain R20291 as a reference strain for comparative purposes. PCR ribotyping was performed as described (*8*). The specific ribotype 027 of each of the clinical isolates was determined by visual analysis and with the GelCompar II v6.5 software (Applied Maths, St-Martens-Latem, Belgium).

Case-patient 1 was a 60-year-old man with a history of coronary disease who required a coronary artery bypass graft because of 3-vessel coronary disease. Forty-eight hours after receiving 3 doses of cefazoline to prevent surgical wound infection, he exhibited severe and diffuse abdominal pain with frequent loose stools (8 bowel movements/day), fever (up to 39°C), and hemodynamic compromise, which required high doses of vasopressors. Stool samples were positive for *C. difficile* by ELISA, and the patient received intravenous metronidazole and oral vancomycin. However, because of the severity of the course of the disease, he underwent an urgent total colectomy with terminal ileostomy. The patient showed progressive improvement, and he was discharged 11 days after surgery. No relapse of *C. difficile* infection was reported in this patient in the next 5 months. Isolation of toxigenic culture and PCR demonstrated that the bacterial pathogen causing the diarrhea was *C. difficile* ribotype 027 (i.e., strain PUC51) (Figure).

**Figure:**
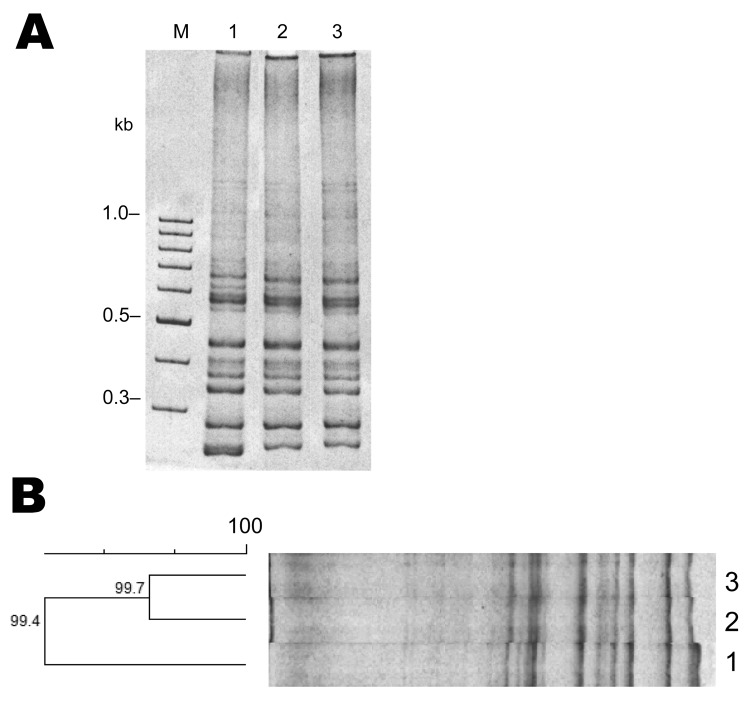
Results of PCR ribotyping of *Clostridium difficile* 027 strains from Chile. M indicates the 100-bp DNA ladder; lane 2, R20291; lane 2, PUC47; lane 3, PUC51. A) PCR ribotyping of *C. difficile* isolates. PCR results show that that the band pattern of the ribosomal intergenic regions of strains PUC47 and PUC51 are similar to those of the reference (epidemic) strain R20291. B) Cluster analysis of strains PUC47, PUC51, and the epidemic strain 027 R20291 shows >99% similarity and that they belong to the same epidemic clade. Scale bar indicates percent identity.

Case-patient 2 was a 46-year-old man with a history of ischemic stroke with hemiparesis of the left side who had experienced a urinary tract infection that had been treated with ciprofloxacin 2 months earlier. Four weeks before admission, he had frequent loose stools with no fever and diffuse abdominal pain after meals. On admission, a computed tomographic scan and angiograph of the abdomen showed pancolitis with colonic wall thickening and scant ascites, suggestive of an inflammatory or infectious cause, without vascular compromise. However, ELISA of stool samples was negative for *C. difficile* toxin. Treatment with ceftriaxone reduced his symptoms, and he was discharged. Seven days after discharge, he had intense diffuse abdominal pain, with frequent loose stools and fever up to 38.9°C, and was again admitted to the hospital. A new computed tomographic scan of the abdomen showed no change; however, an ELISA of a new stool sample for *C. difficile* toxin was positive, and the patient was given oral vancomycin. No relapse of *C. difficile* infection was observed within 3 months of observation. Toxigenic culture from stool samples and PCR identified the *C. difficile* isolate as ribotype 027 (i.e., strain PUC47) (Figure).

Molecular typing analysis showed that both case-patients had a monoclonal infection caused by *C. difficile* ribotype 027. Both isolates had *tcdA*, *tcdB,* and *cdtB* and had a deletion in *tcdC* (data not shown).

In summary, the described severe cases of *C. difficile* infection in Chile were caused by epidemic *C. difficile* ribotype 027. One of these case-patients required urgent colectomy. These results demonstrate that epidemic *C. difficile* 027 strains are present in South America, highlighting the need for enhanced screening for this ribotype in other regions of the continent.
